# VEGF-related polymorphisms identified by GWAS and risk for major depression

**DOI:** 10.1038/tp.2017.36

**Published:** 2017-03-07

**Authors:** T Xie, M G Stathopoulou, F de Andrés, G Siest, H Murray, M Martin, J Cobaleda, A Delgado, J Lamont, E Peñas-LIedó, A LLerena, S Visvikis-Siest

**Affiliations:** 1 UMR INSERM U1122, Interactions Gène-Environnement en Physiopathologie Cardio-Vasculair, Université de Lorraine, Nancy, France; 2 CICAB Clinical Research Centre, Extremadura University Hospital, Badajoz, Spain; 3 Randox Laboratories, Crumlin, UK; 4 Clinica de Rehabilitación de Salud Mental, Servicio Navarro de Salud, Pamplona, Spain; 5 Centro de Atención Primaria Ciudad Jardín, Servicio Extremeño de Salud, Badajoz, Spain; 6 CIBERSAM, Instituto de Salud Carlos III, Madrid, Spain

## Abstract

Depression is a common, severe, disabling mental disease that affects millions of people of all ages worldwide. Various studies have shown that neurotrophic/growth factors have a key role in depression and, more specifically, vascular endothelial growth factor (VEGF) is implicated in the pathogenesis of depression. The purpose of this study was to investigate the potential links between four VEGF-related single-nucleotide polymorphisms (SNPs), previously identified through a genome-wide association study (GWAS) and depression. The direct effects and epistatic interactions of the four VEGF-related SNPs (rs10738760, rs6921438, rs6993770 and rs4416670) on depression were investigated through a case–control study including 437 individuals diagnosed with depression and 477 healthy volunteers as controls. Gender, age and body mass index influence was additionally analyzed. The SNP rs4416670 was associated with increased risk for depression (OR: 1.60, *P*: 0.010). This result demonstrates the existence of relationships between VEGF genetic determinants and depression. This novel association reveals new molecular mechanisms suggesting the potential role of VEGF in depression development that could help to promote a personalized prediction for this severe common disease.

## Introduction

Depression is a common, severe, disabling mental disease that affects millions of people of all ages. Depression is also a leading cause of mortality worldwide, as it can lead to suicide and causes a high burden for patients, their families and the healthcare system. In addition population studies have shown that women are affected by depression more than men, but no study clearly explains the causes of this observation.^1^

Various studies have shown that neurotrophic/growth factors are key factors in depression. One of the most important is brain-derived neurotrophic factor (BDNF) and several studies have focused also on vascular endothelial growth factor (VEGF), which is recognized as a major inducer of angiogenesis and vasculogenesis.^2^ However, VEGF is a multifunctional molecule that has effects also in the central nervous system^3^ VEGF has been shown to have effects on specific areas of the brain, such as hippocampus,^4^ and neurogenesis has been shown to be stimulated *in vitro* and *in vivo* by VEGF.^5^ Proinflammatory cytokines have been implicated in depression,^6, 7^ while VEGF is regulated by proinflammatory cytokines in depression.^8^ Taking together the above information, it has been suggested that interaction between VEGF and proinflammatory cytokines could influence depression and/or the potential efficacy of the treatments with antidepressants.^9^

Numerous studies have investigated the peripheral/serum levels of VEGF in depressed patients and compared them with a control group of healthy subjects; however the results are unclear and ambiguous yet, due to differences in the populations studied. Increased VEGF levels have been observed in depressed patients when compared to healthy controls,^10^ but no differences have been reported in other studies.^11, 12^ In contrast to these results, one study revealed a decrease in plasma and serum VEGF levels in depressed patients.^13^ However, environment factors like gender, age and body mass index (BMI) have not been adequately assessed in the aforementioned association studies and they could potentially explain the controversy observed.^14^

Given the associations between VEGF and depression, a common genetic regulation could be hypothesized. Depression is a complex disease, and it has a heritable background.^15^ Few studies have focused on *VEGF* gene polymorphisms role in depression. Among them, Viikki *et al.*^16^ have shown that one *VEGF* polymorphism, rs699947, is associated with treatment resistant depression. Recently we have identified through a genome-wide association study (GWAS) four single-nucleotide polymorphisms (SNPs) in three chromosomes, which explain almost 50% of the inter-individual variability of VEGF circulating levels.^17^ Therefore, we hypothesize that these strong genetic determinants of VEGF could also be implicated in depression. Thus, the aim of this study was to investigate the direct effects and epistatic interactions of the 4 VEGF-related polymorphisms (rs10738760, rs6921438, rs6993770 and rs4416670) on depression by a case–control study, aiming to detect potential genetic biomarkers between VEGF and depression.

## Materials and methods

### Population

Four hundred thirty seven unrelated Spanish Caucasian patients diagnosed with depressive disorders (132 males/305 females, mean age: 51.49±14.04 years, mean BMI: 28.56±6.56 kg/m^2^) who were attending a Mental Health Centre, were included. The descriptions of depressive disorders and any psychiatric condition, in which depression was a primary symptom and therefore were under antidepressant treatment, were taken from the Diagnostic and Statistical Manual of Mental Disorders (DSM-IV criteria).^18^ A group control of 477 (187 males/290 females, mean age: 66.24±15.01 years, mean BMI: 29.33±11.41 kg/m^2^) Caucasian healthy volunteers from the same geographical region was also studied. The individuals of the control group were selected by psychiatrists who performed a mental and physical revision, as well as revised medical history to check health state, in order to consider about inclusion as healthy individuals. First-degree relatives were also assessed by psychiatrists regarding potential psychiatric disorders. However, to avoid familial stratification issues, only unrelated individuals have been included in the control group. Healthy volunteers with a previous history of adverse drug effects and those with any drug intake in the 2 weeks before the study were excluded from participation in the study. Women who reported or suspected pregnancy were also excluded from the study.

Written informed consent was obtained for all the participants of the study, and the study was performed according to the Helsinki Declaration. This study was approved by the Ethical Committee of Extremadura University Hospital, Badajoz, Spain and of Navarra Health Care System, Pamplona, Spain.

### Genotyping

Five milliliters of peripheral blood were drawn in a tube with EDTA, and then the genomic DNA was isolated with the QIAGEN Blood DNA isolation kit (Qiagen, Hilden, Germany) and evaluated for integrity and concentration through 1% agarose electrophoresis and spectrophotometry. The SNPs rs6921438, rs4416670, rs6993770 and rs10738760 were genotyped using the competitive allele-specific PCR (KASP) chemistry coupled with a FRET-based genotyping system (http://www.lgcgroup.com/services/genotyping/#.VmQwfoR6GFI), and genotyping was confirmed by Randox Laboratories (Crumlin, UK; Evidence Investigator) using an assay based on a combination of multiplex PCR and biochip array hybridization.

### Statistical analysis

Hardy–Weinberg equilibrium was tested using a *χ*^2^-test. The analysis was carried out using the Generalized Multifactor Dimensionality Reduction (GMDR0.9) software (University of Alabama, Birmingham, AL, USA), which is a nonparametric and genetic model-free^19^ alternative to linear or logPage Page 6/istic regression for detecting and characterizing nonlinear interactions among discrete genetic and environmental attributes. Age, gender and BMI were used as co-variables. The score of co-variables was calculated by logistic regression, and then the analysis was performed using the GMDR. Epistatic interactions were assessed only between SNPs with significant direct effects and all the others. The SNPs analyzed are located in different chromosomes; therefore haplotype analysis was not performed as no linkage between SNPs occurs. Direct associations were tested at two-sided *P*<0.012 (corrected for 4 tests) and epistatic interactions were tested at two-sided *P*<0.05/number of final interaction tests.

## Results

The characteristics of the SNPs are presented in Table 1. Hardy–Weinberg equilibrium was not observed in the SNP rs4416670 in the group of cases due to significant disagreement found between expected and observed frequencies, thus indicating a difference in the frequency of this SNP in depression patients.

### Associations of the SNPs in cases-controls

Among the four SNPs studied, only the rs4416670 was significantly associated with depression. As shown in Tables 2A and 2B, this SNP was associated with an increased risk for depression (OR: 1.60; Table 2A), while the minor allele C was more frequent in patients (Table 2B). No association with depression was found for the other three SNPs.

### Epistatic interactions between the 4 SNPs and depression

As only one SNP (rs4416670) was directly associated with depression, we have assessed only the epistatic interactions involving this SNP. The significance cut off value was set at *P* 0.05/3 (3 interaction tests)=0.016.

No significant interaction was identified.

## Discussion

In this study, we identified links between VEGF-related genetic polymorphisms and depression. More precisely, one SNP (rs4416670) related to VEGF levels^17^ was associated with increased risk for depression. The minor allele of this polymorphism has been previously associated with decreased VEGF levels.^17^ As this polymorphism has a very high frequency (almost 50%), the effect of the minor allele can affect a large percentage of the population, thus having an important impact for public health.

Several studies revealed that proinflammatory cytokines are implicated in the etiology of depression.^6, 20^ Miller *et al.*^20^ have underlined the relationship between proinflammatory cytokines and depression in individuals with disorders that include chronic inflammation. We have previously shown that in supposed healthy individuals the C allele of rs4416670 was positively associated with l-selectin mRNA, and that VEGF_145_ mRNA positively regulated l-selectin expression.^21^ At the same time, l-selectin has been shown to correlate with mental diseases such as the pathogenesis of schizophrenia^22^ and the severity of panic disorder,^23^ thus indicating that this molecule can play a significant role in these conditions. Despite these associations have not been demonstrated yet in patients with depression, based on our results, we could propose the hypothesis that l-selectin could be implicated in depression development, and this effect could be additionally dependent on the genotype of rs4416670. The presence of the C allele of this SNP is increasing expression of l-selectin, which could indicate an increased risk for depression. This hypothesis should be tested in future studies.

Concerning the role of VEGF, the C allele of the rs4416670 (currently linked with increased risk of depression) was associated with decreased levels of VEGF.^17^ Therefore, our present findings are consistent with a previous study showing a negative correlation between serum VEGF concentration and scores on a widely used diagnostic measure of depression, the Hamilton Depression Rating Scale.^12^ Furthermore, a significantly decrease in plasma VEGF has been shown in individuals exposed to a stressful military training and environment.^24^ Thus, it is possible that the present findings could be also explained in the frame of the diathesis-stress model of depression,^25, 26^ making individuals carrying this VEGF SNP more likely to develop depression due to a significant downregulation of VEGF blood levels during or after exposure to stress, and/or to a greater vulnerability to stress. Another previous study also showed that the subset of patients who responded to antidepressant treatment were those with decreased plasma VEGF levels.^27^ Therefore, future studies will have to test whether these SNPs show less tolerance to environmental stress, and/or decreases in plasma VEGF levels and whether they are overrepresented among depressive patients who respond to antidepressant treatment contrary to those who do not respond.

In conclusion, to our knowledge, this is the first study that has assessed the common genetic regulation between VEGF and depression with important original results that reveal novel molecular mechanisms for this common, severe and disabling mental disease. The identification of SNPs that may predict susceptibility to depression and/or response to treatment can have important implication in clinical practice. Further research on these mechanisms involved in depression development and response to treatment, as well as the functional role of these SNPs could allow the implementation of these findings in personalized risk prediction and hopefully targeted treatment of depression.

## Figures and Tables

**Table 1:** SNPs characteristics in cases and controls.

*Chr*	*SNP*	*Minor allele*	*MAF* *patients*	*HWE* *patients*	*MAF* *control*	*HWE* *control*
6	rs4416670	C	0.469	0.035	0.468	0.579
6	rs6921438	A	0.408	0.496	0.454	0.606
8	rs6993770	T	0.356	0.412	0.340	0.663
9	rs10738760	A	0.463	0.076	0.470	0.769

Abbreviations: Chr, chromosome; HWE, Hardy–Weinberg equilibrium; MAF, minor allele frequency; SNP, single-nucleotide polymorphism.

**Table 2A:** Associations between VEGF-related with depression

*Marker*	*Allele Major/minor*	*OR (95% CI)*	*P*	*Chi-square*
rs4416670	T/C	1.60 (1.03–2.46)	0.010	4.38 (*P*=0.036)

Abbreviations: CI, confidence interval; OR, odds ratio; VEGF, vascular endothelial growth factor.

**Table 2B:** Alleles investigation for the rs4416670 in patients versus controls

*Marker/allele*	*Combination score*^a^		*Class level*^b^
	*Patient (*n=*437)*	*Control (*n=*477)*	
*rs4416670*			
CC	56.39	47.00	1
TC	119.36	109.75	1
TT	41.93	58.79	0

a Probability of being affected.

b 0—control; 1—patient.
